# Measurement of Oral Epithelial Thickness by Optical Coherence Tomography

**DOI:** 10.3390/diagnostics9030090

**Published:** 2019-08-06

**Authors:** Dario Di Stasio, Dorina Lauritano, Hasan Iquebal, Antonio Romano, Enrica Gentile, Alberta Lucchese

**Affiliations:** 1 Multidisciplinary Department of Medical-Surgical and Dental Specialties, University of Campania—Luigi Vanvitelli, 80138 Naples, Italy; 2 Department of Medicine and Surgery, Centre of Neuroscience of Milan, University of Milano-Bicocca, 20126 Milan, Italy; 3 ECU School of Dental Medicine, 1851 MacGregor Downs Road, Greenville, NC 27834, USA

**Keywords:** in vivo, oral mucosa, optical coherence tomography

## Abstract

Optical coherence tomography (OCT) is a real-time, in-situ, non-invasive imaging device that is able to perform a cross-sectional evaluation of tissue microstructure based on the specific intensity of back-scattered and reflected light. The aim of the present study was to define normal values of epithelial thickness within the oral cavity. OCT measurements of epithelial thickness were performed in 28 healthy patients at six different locations within the oral cavity. Image analysis was performed using Image J 1.52 software. The healthy epithelium has a mean thickness of 335.59 ± 150.73 µm. According to its location within the oral cavity, the epithelium showed highest values in the region of the buccal mucosa (659.79 µm) and the thinnest one was observed in the mouth’s floor (100.07 µm). OCT has been shown to be useful for the evaluation of oral mucosa in vivo and in real time. Our study provides reference values for the epithelial thickness of multiple sites within the oral cavity. Knowledge of the thickness values of healthy mucosa is, therefore, of fundamental importance.

## 1. Introduction

Optical coherence tomography (OCT) is an imaging diagnostic device which measures the intensity of backscattered and reflected light so that it can produce high-resolution microscopic images of biological tissues [1], with an axial and lateral resolution estimated, respectively, at 13–17 and 17–22 µm [2].

OCT usually has an interferometer-based operating mechanism, with a low-coherence-length broadband light source. A low-coherence diode produces a light beam of variable wavelength in the infrared spectrum and it is conveyed by an optical probe. The initial light beam is divided into two: an optic fiber splitter between the biological tissue and reference arms of a Michelson or Mach–Zehnder interferometer. The two light beams converge again in the optical fiber after being diffused backwards from the tissue and reflected by the reference mirror, thus causing interference. A photodetector or spectrometer (depending on the type of OCT) registers this interference, transforms the signal from a luminous to a digital one and scans its information: finally, a computer receives the signal for graphical processing. According to the structure of the reference arm optics, OCT can be classified into time domain (TD) and frequency domain (FD)-OCT [3,4].

The TD-OCT or time-domain OCT (as it measures the time the light takes to be reflected) is an early and simple device but it allows the creation of approximately 400 axial scans per second [1]. According to the different components they employ, OCT devices can provide several types of analysis.

In particular, three types of microscopic exploration can be produced by OCT technology:

1. the A-scan type, which uses a single light beam and defines the depth of the findings observed thanks to their reflectivity;

2. the B-scan type, which, on the contrary, employs multiple A-scans on the x- and z-axes that provide a cross-sectional 2D view of the observed structure and the obtained image is also called longitudinal;

3. the C-scan type, which is also a 2D cross-sectional view on the x- and y-axes, but the image obtained is called “en face’’;

4. Matching a series of B-scan and C-scan images, it is possible to create a 3D reconstruction of the observed structures [1].

The FD-OCT is divided into swept-source (SS) and spectral domain (SD)-OCT according to the output characteristics and the structure of the light source receiving components [5]. The hardware characteristics from the light source, the spectrometer, and the optical components of FD-OCT inevitably create nonlinearity in the k-domain [6]. This nonlinearity to the k-domain diminishes the depth resolution in the FD-OCT system using the inverse Fourier transform relationship of the interference pattern. Several compensation methods have been created to overcome this nonlinearity [7]. One of these consists of inserting the interferometer into the SS-OCT system, receiving the linearized interference pattern to the k-domain and using the pattern as a trigger signal [8]. SS-OCT, compared to other OCT technologies, is faster and consequently widely used because of its speed [9,10]. Moreover, SS-OCT devices have shown an improved sensitivity roll-off and attenuation of OCT signal, and can detect more structural signals from deeper tissues compared to SD-OCT [11]. 

Previous investigations demonstrated a progressive thickening from normal epithelium through the different grades of dysplasia to early invasive carcinoma in vocal fold lesions [12] and in the oral cavity [13]. OCT images provided microanatomic information about the epithelium, basement membrane, and lamina propria, and have proved to be a valid and practicable method for the determination of epithelial thickness in vivo [14,15,16]. There are also other devices for in the vivo imaging of the oral mucosa such as high-frequency ultrasonography (US) [17]. A direct comparison between OCT and high-frequency ultrasound (US) [18,19] highlighted that the spatial resolution of OCT is significantly better.

On this basis, the aim of the present study was to measure normal values of epithelial thickness within the oral cavity by SS-OCT.

## 2. Materials and Methods

A total of 28 patients, referred to the Oral Pathology Unit at the University of Campania, “Luigi Vanvitelli”, in Naples (Italy), were selected to evaluate the epithelial thickness of the oral mucosa. All subjects gave their informed consent for inclusion before they participated in the study. The Ethical Committee of the same university approved the study (n.0000689/2014, approved on 4 September 2014). Inclusion criteria were healthy volunteers with clinically normal mucous membranes, who did not report any other systemic diseases, non-smokers and aged over 18. Pregnant women were excluded from this study. 

**Figure 1:**
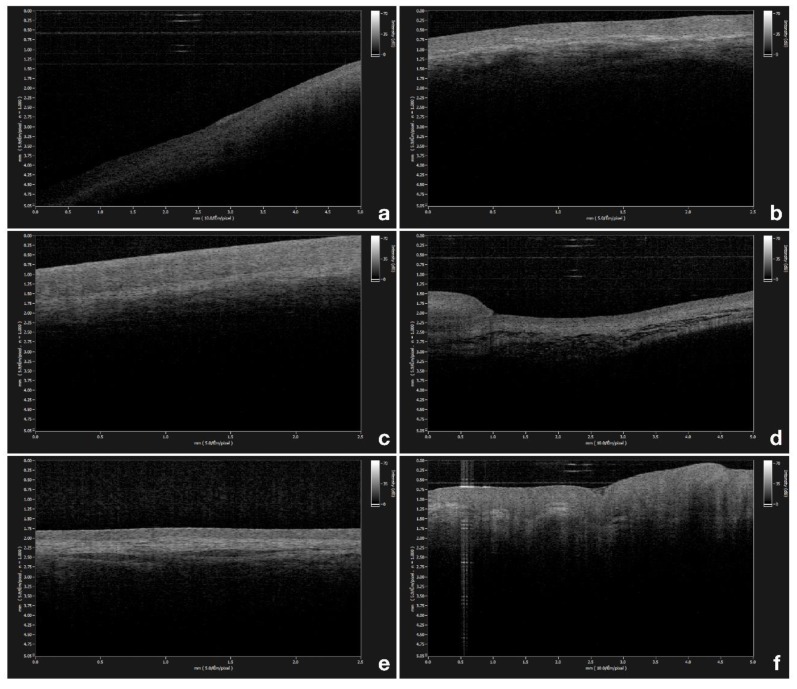
(**a**) Optical coherence tomography (OCT) image of healthy gingiva; (**b**) OCT image of healthy labial mucosa; (**c**) OCT image of healthy buccal mucosa; (**d**) OCT image of healthy ventral surface of the tongue; (**e**) OCT image of healthy floor of the mouth; (**f**) OCT image of healthy tongue dorsum.

OCT measurements of epithelial thickness were performed at the exact same site in all patients, bilaterally at 6 different locations within the oral cavity, namely, the gingiva (Figure 1a), the labial mucosa (Figure 1b), the buccal mucosa (Figure 1c), the ventral surface of the tongue (Figure 1d), the floor of the mouth (Figure 1e), and the tongue dorsum (Figure 1f). Three repeated scans were performed in the same location.

### 2.1. Epithelial Thickness Mesurement

Image analysis was performed using Image J 1.52 software [20]. Images were processed in order to obtain the measurements of epithelial thickness. 

The measurements were carried out as follows: 1. Open ImageJ software. 2. Drag and drop the picture into the software interface. 3. A segment was drawn along the ruler at the left side margin of the image using the “straight line” tool provided by the software. The examiners drew a distance of 0.5 mm along the ruler. The software calculates the distance in pixels of the selected segment. 4. Analyze menu > set scale > known distance (500) > unit of length (µm). The software automatically recalculates the number of pixels/mm (0.088 pixels/µm). 5. Epithelial thickness outline was created using the “straight line” tool and tracing a line starting from the most recognizable superficial point of epithelium up to the recognizable signal change in terms of gray scale with the computer mouse in cases of desktop computers or trackpad in cases of laptops; this is made possible by the fact that the epithelium reveals a less intense signal than the lamina propria in the B-mode OCT scan. 6. Analyze menu > measure. The length in µm was then calculated.

Two different operators performed the computerized measurements; repeated measurements to minimize errors have been made. The two authors were blinded to the readings of the other authors. Just the lead author had access to the results of the measurements. One-way ANOVA has been used to highlight differences between repeated measurements between the left and right side of the oral cavity.

### 2.2. OCT System

Optical coherence tomography (OCT) is a non-invasive imaging device that can be employed for both real-time and in-situ investigations, able to perform a cross-sectional evaluation of tissue microstructure based on the specific intensity of back-scattered and reflected light [21]. This study used a swept-source OCT instrument (IVS-300) by Santec™, with a grip type probe of the same manufacturer directly applied to the oral mucosa after application of ultrasound gel with a working distance of 10 mm. Centre wavelength was 1310 ( ± 30) nm, axial resolution of the system was ≤ 12 µm (in tissue); lateral resolution was 22 µm. Both 2D and 3D imaging modes were used by the default native software Inner Vision™ (Windows 7 OS based) and a native OCT viewer elaborated all images. The scan mode was set for 5 × 5mm on the X and Y axis and a pixel size of 500 and Z offset 24 (the number of the pixels to shift the A-lines of the frame to correct for the path length difference between the OCT sample arm and reference arm in air) [22]. 

### 2.3. Statistical Analysis

Demographic parameters and the epithelial thickness of each location were collected and analyzed through descriptive statistics. Kurtosis and skewness were employed to further evaluate the distribution of the measured values of epithelial thickness. Differences between the six different anatomical locations were determined by the non-parametric Mann–Whitney U test. The software R was used for all computations and p values ≤ 0.05 were considered statistically significant.

## 3. Results

A total of 504 OCT images per side from six different locations within the oral cavity were obtained from 28 white healthy volunteers with a mean age of 35.57 years (range, 20–59 years) and a sex distribution of 35.7% men and 64.3% women.

**Figure 2:**
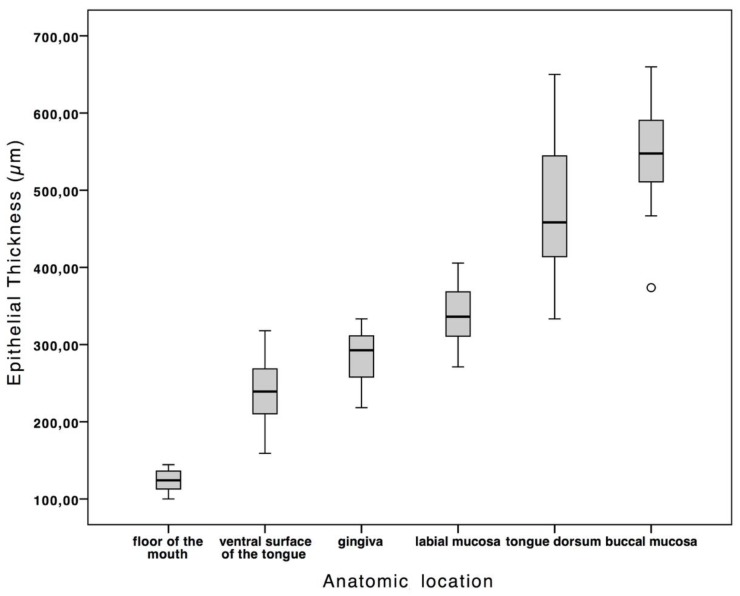
The box plot shows the normal values of epithelial thickness according to anatomic location. The difference in epithelial thickness between the different anatomical locations was statistically significant—*p* ≤ 0.05.

According to its location within the oral cavity, the epithelium showed a variable thickness. Details regarding measured sites are reported in Table 1. The highest and the thinnest values were observed, respectively, in the region of the buccal mucosa, −659.79 µm, and in the mouth’s floor, −100.07 µm. The difference in epithelial thickness between the different anatomical locations was statistically significant—*p* ≤ 0.05 (Figure 2). The healthy epithelium has a mean thickness of 335.59 ± 150.73 µm. There were no significant differences between the left and right side of the oral cavity, as the ANOVA analysis of the measurements per image underlined. Therefore, thanks to the repeated measurements, it is possible to exclude an interobserver variability in this study. 

Through the OCT examination of a healthy buccal mucosa, it was possible to clearly establish the stratified squamous epithelium (SS) along the mucosal surface, underlying the lamina propria (LP), and the transition between these tissues along the basement membrane (BM) boundary. 

The appearance of these tissues is strictly related to their optical density and scattering properties; that is why the lower optical density of buccal mucosa epithelium is darker, as evident in a lower signal intensity. Conversely, the optically denser LP appears brighter than the BM one, caused by a higher signal intensity. The lower contrast is a typical characteristic of a keratinized epithelium. Moreover, there are very few differences between the OCT image of the gingival mucosa and its own lamina (LP) at a depth of 285.04 ± 32.98 µm.

## 4. Discussion

In vivo, non-invasive imaging techniques such as magnetic resonance imaging [9], confocal laser microscopy [23,24,25] and ultrasound imaging [17] have found widespread applications in medicine. Each of these techniques measures a different physical property and has a resolution and penetration range that prove advantageous for specific applications. One of the main limitations of this technique is that the quality of the images remains strongly user dependent [1] and the large, rigid probe, which makes examination of the posterior and less-accessible locations of the oral cavity difficult [14]. 

The literature has shown that OCT is a valid method for determining epithelial thickness in vivo [15,26]. With this technique, indeed, it is possible to perform the non-invasive cross-sectional imaging of biological tissues by measuring their optical reflections [9]. Therefore, it is essential to know the dimensions of the different layers of normal mucosal tissue in different anatomical sites within the oral cavity in order to consider OCT a valuable imaging technique for analyzing the oral mucosa. Within the oral cavity, healthy epithelium varies from 75 to 550 µm in thickness depending on the anatomic site [27,28]. Prestin et al. [14] were the first to provide reference values for epithelial thickness within the oral cavity. In the present study, we presented data on the epithelial thickness of additional oral cavity sites compared to those presented by Prestin et al.

Furthermore, Tsai et al. [13] studied four groups of oral mucosa samples, namely, healthy mucosa, epithelial hyperplasia, moderate dysplasia and squamous cell carcinoma of the oral cavity (OSCC), combining the results obtained with histology. The authors showed the analysis and statistical results of the SS-OCT scan images using three diagnostic indicators (the standard deviation of a scan signal profile in mode A, the exponential decay constant of a spatial scanning frequency spectrum in mode A and epithelial thickness). The obtained data revealed that in abnormal oral mucosa samples, the standard deviation becomes larger, the spatial frequency spectrum decay constant becomes smaller and the epithelium becomes thicker.

In addition, Kraft et al. [29] were able to demonstrate a progressive thickening from normal epithelium through the different grades of dysplasia to early invasive carcinoma in vocal fold lesions. Hence, with the help of OCT, the grade of dysplasia can be determined approximately by morphometric measurement of epithelial thickness. 

On the basis of this, the thickness of the epithelium and the integrity of the basement membrane, identifiable with the aid of OCT, could be a valid aid for the clinician to obtain information on the possible nature and grade of the dysplasia of oral cavity lesions.

## 5. Conclusions

OCT has been shown to be a simple method for the evaluation of oral mucosa in vivo. Our study provides reference values for the epithelial thickness of multiple sites within the oral cavity. As evidenced by the literature, increased epithelial thickness values may be related to pathological changes. Knowledge of the thickness values of healthy mucosa is, therefore, of fundamental importance for this purpose. Excisional biopsy followed by histological examination is still the gold standard for a reliable assessment of oral cavity lesions. Despite the limitations of this study, due in part to the sample size, the present data allow us to consider OCT as a promising technique to detect and monitor different pathological conditions and have the potential to provide an in-vivo, real-time, non-invasive method for the early diagnosis of cancer and precancer.

## Figures and Tables

**Table 1:** Values of epithelial thickness within the oral cavity.

Anatomic Location		Thickness (µm)	SD
gingiva	Mean	285.04	± 32.98
	Min	218.30	
	Max	333.33	
labial mucosa	Mean	339.83	± 36.44
	Min	271.19	
	Max	405.56	
buccal mucosa	Mean	545.40	± 62.45
	Min	373.75	
	Max	659.79	
ventral surface of the tongue	Mean	239.79	± 37.30
	Min	159.09	
	Max	318.00	
floor of the mouth	Mean	124.09	± 13.53
	Min	100.07	
	Max	144.44	
tongue dorsum	Mean	479.32	± 83.56
	Min	333.33	
	Max	650.02	
